# A European Multicenter Outcome Study of Perioperative Airway Management Policies following Midface Surgery in Syndromic Craniosynostosis

**DOI:** 10.1097/PRS.0000000000011317

**Published:** 2024-01-30

**Authors:** Iris E. Cuperus, Irene M. J. Mathijssen, Marie-Lise C. van Veelen, Anouar Bouzariouh, Ingrid Stubelius, Lars Kölby, Christopher Lundborg, Sumit Das, David Johnson, Steven A. Wall, Dawid F. Larysz, Krzysztof Dowgierd, Małgorzata Koszowska, Matthias Schulz, Alexander Gratopp, Ulrich-Wilhelm Thomale, Víctor Zafra Vallejo, Marta Redondo Alamillos, Rubén Ferreras Vega, Michela Apolito, Estelle Vergnaud, Giovanna Paternoster, Roman H. Khonsari, Workgroup Craniosynostosis, European Reference Network CRANIO

**Affiliations:** Rotterdam, the Netherlands; Gothenburg, Sweden; Oxford, United Kingdom; Olsztyn, Poland; Berlin, Germany; Madrid, Spain; and Paris, France; From the Departments of 1 Plastic and Reconstructive Surgery and Hand Surgery; 2 Neurosurgery; 3 Anaesthesiology, Erasmus University Medical Center; Departments of 4 Anesthesiology and Intensive Care; 5 Plastic Surgery, University of Gothenburg, Sahlgrenska University Hospital; Departments of 6 Paediatric Anaesthesia; 7 Plastic and Reconstructive Surgery, John Radcliffe Hospital; 8 Department of Head and Neck Surgery for Children and Adolescents, Regional Specialized Children’s Hospital Popowski; 9 Department of Head and Neck Surgery for Children and Adolescents, University of Warmia and Mazury; Departments of 10 Pediatric Neurosurgery; 11 Pediatric Pulmonology, Immunology, and Intensive Care Medicine, Charité-Universitätsmedizin Berlin, corporate member of Freie Universität Berlin and Humboldt-Universität zu Berlin; Departments of 12 Oral and Maxillofacial Surgery; 13 Anaesthesiology, University Hospital 12 de Octubre; Departments of 14 Maxillofacial Surgery and Plastic Surgery; 15 Pediatric and Obstetrical Anesthesiology and Reanimation; 16 Pediatric Neurosurgery, Hôpital Necker-Enfants Malades, AP-HP; 17 National Reference Center for Craniosynostosis and Craniofacial Malformations (CRANIOST); 18 Faculty of Medicine, Paris Cité University.

## Abstract

**Background:**

Perioperative airway management following midface advancements in children with Apert and Crouzon–Pfeiffer syndromes can be challenging, and protocols often differ. This study examined airway management following midface advancements and postoperative respiratory complications.

**Methods:**

A multicenter, retrospective cohort study was performed to obtain information about the timing of extubation, perioperative airway management, and respiratory complications after monobloc or Le Fort III procedures.

**Results:**

A total of 275 patients (monobloc surgery, *n* = 129; Le Fort III surgery, *n* = 146) were included. Sixty-two patients received immediate extubation and 162 received delayed extubation; 42 had long-term tracheostomies, and 9 had perioperative short-term tracheostomies. In most centers, short-term tracheostomies were reserved for selected cases. Patients with delayed extubation remained intubated for 3 days (interquartile range, 2 to 5 days). The rate of no or only oxygen support after extubation was comparable between immediate and delayed extubation groups (58 of 62 patients [94%] and 137 of 162 patients [85%], respectively). However, the immediate extubation group developed fewer cases of postoperative pneumonia than did the delayed group (0 of 62 [0%] versus 24 of 161 [15%]; *P* = 0.001). Immediate extubation also appeared safe in moderate to severe obstructive sleep apnea, as 19 of 20 patients (95%) required either no or only oxygen support after extubation. The odds of developing intubation-related complications increased by 21% with every extra day of intubation.

**Conclusions:**

Immediate extubation following midface advancements was found to be a safe option, as it was not associated with respiratory insufficiency but did lead to fewer complications. Immediate extubation should be considered routine management in patients with no or mild obstructive sleep apnea, and should be the aim in moderate to severe obstructive sleep apnea cases after careful assessment.

**CLINICAL QUESTION/LEVEL OF EVIDENCE:**

Therapeutic, III.

Apert and Crouzon–Pfeiffer syndromes are two forms of syndromic craniosynostosis characterized by multiple suture synostosis, midface hypoplasia, and exorbitism. As a result of their midface hypoplasia, two-thirds of these children are affected by obstructive sleep apnea (OSA).^1^ Other potential airway-obstructing abnormalities in these children include choanal atresia, nasal septum deviation, hypertrophic adenoid or tonsils, mandibular hypoplasia, palatal deformities, and tracheal cartilage anomalies.^2–5^ Upper airway endoscopy shows that there are often multilevel airway obstructions in these children, and children without OSA can also have partial obstructions.^6,7^

Midface hypoplasia and exorbitism can be treated surgically with a monobloc procedure, Le Fort III, a variant of these procedures (such as Le Fort II surgery with simultaneous zygomatic repositioning), or facial bipartition, and surgery is usually combined with distraction.^8^ Blood loss during these advancements can be excessive, and transfusion is generally needed.^9^ The difficult airway, high prevalence of OSA, profuse intraoperative bleeding with high transfusion rates, and the anticipated postoperative swelling of the upper airway cause anesthesiologic concerns following midface advancements in these children.^9–13^

Little is known about the safety of immediate extubation and (respiratory) complications after midface advancements. Standard operating procedures (SOPs) differ among hospitals and often lack scientific foundations. This multicenter study aimed, first, to examine airway management following midface advancement with regard to respiratory outcome and complications in children with Apert and Crouzon–Pfeiffer syndromes within several European centers of expertise; and second, to formulate principles for a collective SOP for perioperative airway management following midface surgery in these children.

## PATIENTS AND METHODS

### Participating Craniofacial Centers of Expertise

Given the study’s retrospective nature, it was exempted from review by the Dutch institutional research ethics committee. University Hospital 12 de Octubre (Madrid, Spain), Sahlgrenska University Hospital (Gothenburg, Sweden), Oxford Craniofacial Unit (Oxford, United Kingdom), Charité University Hospital (Berlin, Germany), Provincial Specialist Children’s Hospital (Olsztyn, Poland), Hôpital Necker-Enfants Malades (Paris, France), and Erasmus Medical Centre (Rotterdam, the Netherlands), all members of the European Reference Network CRANIO, participated in this multicenter study.

### Outcome Variables and Data Collection

All children with Apert or Crouzon–Pfeiffer syndrome who had undergone a midface advancement between December of 1983 and August of 2022 (ie, monobloc surgery, facial bipartition, Le Fort III, or variant) were eligible for inclusion. The center’s inclusion periods could vary based on the availability of older patient files. Patients were split into 4 groups based on perioperative airway treatment: the immediate extubation group comprised patients who were extubated directly after surgery in the operating room; the delayed extubation group comprised those who received prolonged intubation and were extubated later in the intensive care unit (ICU); the short-term/elective tracheostomy group comprised patients who were tracheostomized to bridge surgery; and the long-term tracheostomy group comprised patients who were tracheostomized in case of severe OSA and not only to bridge surgery.

Primary outcomes were respiratory support after extubation or cessation of mechanical ventilation in tracheostomy patients and postoperative pneumonia. Secondary outcomes were type of postoperative respiratory support and complications. Demographic data (sex, diagnosis, age, OSA); surgical data (type of surgery, indication for surgery, intraoperative estimated blood loss, intraoperative transfused blood volume); anesthesiologic data (timing of extubation, numbers of days intubated, indication for tracheostomy or delayed extubation, respiratory support before and after extubation); and postoperative (respiratory) complications were collected from patients’ files. Files were scanned until 1 month postoperatively for complications. The collected intubation-related complications were pneumonia and pressure ulcers.^14–17^ The obstructive apnea-hypopnea index (oAHI) score obtained from polysomnography was used to classify OSA, as follows: for no or mild OSA, oAHI score less than 5; for moderate OSA, oAHI score of 5 or higher and less than 10; and for severe OSA, oAHI score of 10 or higher.

### Surgical Treatment

The type of midface advancement was determined by individual facial abnormalities, the presence of increased intracranial pressure or need for additional forehead advancement, the surgeon’s preference and expertise, and the patients’ or parents’ wishes. Given the severity of midface hypoplasia in both syndromes, there is often an indication for distraction.

### Statistical Analysis

Data were imported into R statistical software (version 4.1.2; R Foundation for Statistical Computing) for analysis. Histograms and QQ-plots were evaluated to assess the distribution of continuous variables. Normally distributed continuous data are presented as means with SD, and skewed data as medians with interquartile ranges (IQRs). Categorical data are presented as counts and proportions.

A chi-square test of independence was performed to assess the relationship between respiratory support after extubation or postoperative pneumonia and immediate or delayed extubation. A Bonferroni correction was used to correct for multiple testing, and a *P* value of 0.05/2 = 0.025 was considered statistically significant. Due to the small sample size of patients with tracheostomies, no statistical analysis was performed between patients with short-term and long-term tracheostomies, and only descriptive statistics were provided. Logistic regression was used to investigate the effect size of duration of intubation/mechanical ventilation in intubation-related complications, corrected for OSA and transfused blood volume (mL/kg). The multiple-imputation-by-chained equation (MICE package) was used to handle missing values. An odds ratio with a corresponding 95% confidence interval was calculated. Detailed information on the multiple imputation method used can be found in the Supplemental Digital Content. (**See Appendix, Supplemental Digital Content 1**, which shows detailed information on the multiple imputation used to handle missing data, http://links.lww.com/PRS/H214.) The Hosmer–Lemeshow test (ResourceSelection package) was used to test the goodness of fit of the model.

## RESULTS

### Study Population

**Fig. 1.**
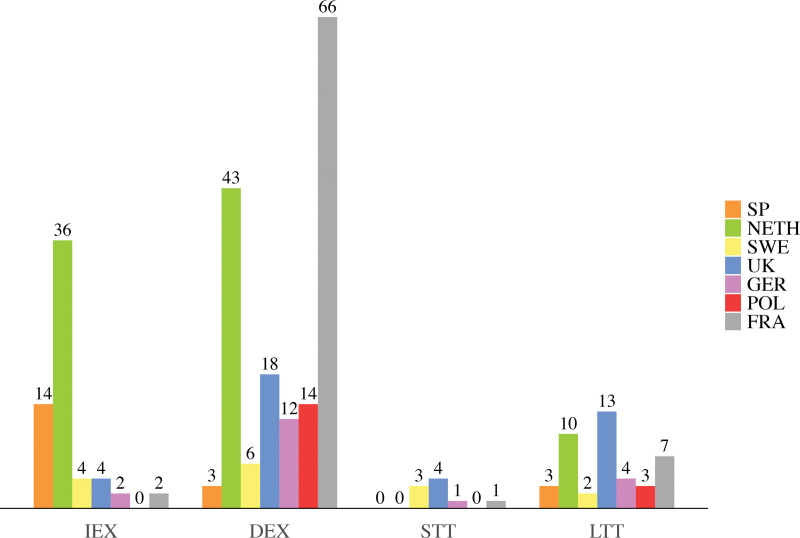
Bar chart showing absolute numbers of patients per perioperative airway management strategy and medical center location. *IEX*, immediate extubation; *DEX*, delayed extubation; *STT*, short-term tracheostomy; *LTT*, long-term tracheostomy; *SP*, Spain; *NETH*, the Netherlands; *SWE*, Sweden; *UK*, United Kingdom; *GER*, Germany; *POL*, Poland; *FRA*, France.

Eventually, 275 patients, with 129 monobloc and 146 Le Fort III procedures, were included. Sixty-two patients received immediate extubation, 162 received delayed extubation, 9 received short-term tracheostomies, and 42 received long-term tracheostomies (Table 1). Twenty patients from Spain, 89 from the Netherlands, 15 from Sweden, 39 from the United Kingdom, 19 from Germany, 17 from Poland, and 76 from France were included (Fig. 1). The inclusion periods were from 2011 to 2022 for Spain, 1983 to 2022 for the Netherlands, 2000 to 2022 for Sweden, 1984 to 2021 for the United Kingdom, 2013 to 2021 for Germany, 2012 to 2022 for Poland, and 2011 to 2022 for France.

**Table 1.** Patient Characteristics

	Timing of Extubation/Tracheostomy			
	IEX	DEX	STT	LTT
No. of patients	62	162	9	42
Male:female, no. (%)	32:30 (52; 48)	72:90 (44; 56)	3:6 (33; 67)	22:20 (52; 48)
Median age (IQR), yrs	8.6 (5.6–14.1)	8.6 (4.0–14.1)	8.5 (4.0–10.2)	4.5 (2.0–7.0)
Diagnosis, no. (%)				
Apert	17 (27)	51 (31)	2 (22)	7 (17)
Crouzon–Pfeiffer	45 (73)	111 (69)	7 (78)	35 (83)
OSA classification,^a^ no. (%)				
Normal/mild	41 (66)	85 (53)	1 (11)	-
Moderate	4 (6)	25 (15)	2 (22)	4 (10)
Severe	16 (26)	37 (23)	4 (45)	37 (84)
Unknown	1 (2)	15 (9)	2 (22)	1 (6)
Type of surgery, no. (%)				
Monobloc	38 (61)	61 (38)	5 (56)	25 (59)
Le Fort III	24 (39)	101 (62)	4 (44)	17 (41)
Median EBL^b^ (IQR), mL/kg	40 (24–60)	43 (28–70)	120 (79–123)	88 (49–231)
Median TBV^c^ (IQR), mL/kg	17 (8–25)	23 (11–43)	24 (7–31)	35 (24–69)

IEX, immediate extubation; DEX, delayed extubation; STT, short-term tracheostomy; LTT, long-term tracheostomy; EBL, estimated blood loss; TBV, transfused blood volume.

a For 1 patient in the IEX group, 15 in the DEX group, 2 in the STT group, and 1 in the LTT group, the OSA classification was unknown.

b For 10 patients in the IEX group, 110 in the DEX group, 6 in the STT group, and 28 in the LTT group, the estimated blood loss was unknown.

c For 7 patients in the IEX group, 28 in the DEX group, 3 in the STT group, and 5 in the LTT group, the transfused blood volume was unknown.

### Considerations in Perioperative Airway Management Strategy

**Fig. 2.**
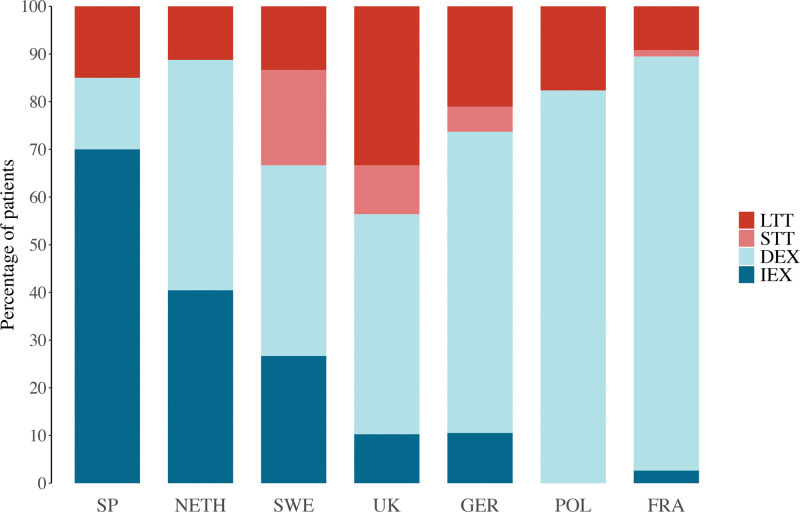
Stacked bar chart showing distribution of perioperative airway management strategies by medical center location. *IEX*, immediate extubation; *DEX*, delayed extubation; *STT*, short-term tracheostomy; *LTT*, long-term tracheostomy; *SP*, Spain; *NETH*, the Netherlands; *SWE*, Sweden; *UK*, United Kingdom; *GER*, Germany; *POL*, Poland; *FRA*, France.

In most centers, the majority of patients received delayed extubation (Fig. 2). Immediate extubation was routinely used in centers in Spain and the Netherlands. Those centers have changed their protocol to immediate extubation in the last 10 years after experience gained from their anesthesiology teams. Short-term tracheostomies were used in 4 of 7 centers (in Sweden, the United Kingdom, Germany, and France), and currently, the center in the United Kingdom has moved to regular use of short-term tracheostomy to ensure a straight airway in case of the need for imaging under general anesthesia or readjustment of the frame during the distraction period. Detailed, center-specific considerations can be found in the Supplemental Digital Content. (**See Table, Supplemental Digital Content 2**, which shows a summary of center-specific considerations and protocols in airway management following midface advancement [*IEX*, immediate extubation; *DEX*, delayed extubation; *STT*, short-term tracheostomy; *LTT*, long-term tracheostomy; *SP*, Spain; *NETH*, the Netherlands; *SWE*, Sweden; *UK*, United Kingdom; *GER*, Germany; *POL*, Poland; *FRA*, France], http://links.lww.com/PRS/H215.) The primary reported indications for delayed extubation or prolonged mechanical ventilation in tracheostomy patients were routine management in all midface procedures in 144 of 200 patients (72%) and severe OSA in 28 of 200 (14%) (Table 2). In 3 of 200 patients (2%), the indication for delayed extubation was failed attempts at the end of surgery. All 3 patients were successfully extubated after 1 day.

**Table 2.** Reported Indications for Delayed Extubation or Prolonged Mechanical Ventilation in Patients with Tracheostomies by Medical Center^a^

	Craniofacial Center							Total
	Spain	Netherlands	Sweden	UK	Germnny	Poland	France	
Routine management in all midface surgery procedures	1 (33)	42 (88)	2 (22)	13 (41)	13 (76)	16 (94)	57 (77)	144 (72)
Severe OSA	0 (0)	0 (0)	3 (33)	13 (41)	1 (6)	1 (6)	10 (14)	28 (14)
Severe perioperative EBL	0 (0)	2 (4)	0 (0)	0 (0)	0 (0)	0 (0)	0 (0)	2 (1)
Failed attempt to extubate	0 (0)	2 (4)	0 (0)	0 (0)	0 (0)	0 (0)	1 (1)	3 (2)
Severe postoperative swelling	0 (0)	0 (0)	3 (33)	0 (0)	0 (0)	0 (0)	5 (7)	8 (4)
Other/not reported	2 (67)	2 (4)	1 (12)	6 (18)	3 (18)	0 (0)	1 (1)	15 (7)

EBL, estimated blood loss.

a Values represent number of patients (percentage).

Regarding immediate extubation, 50 of 60 cases (81%) occurred in Spain or the Netherlands. In the other centers, it seemed to be reserved for selected cases, such as in patients who were slightly older (minimum age of 5.8 years); had small amounts of estimated blood loss (median, 30 mL/kg) or transfused blood volume (median, 8.1 mL/kg); and, apart from the center in the United Kingdom, did not have moderate to severe OSA.

### Respiratory Support before Extubation

Patients with delayed extubation remained intubated for 3 days (IQR, 2 to 5 days). Most (86%) received mechanical ventilation during intubation (Table 3). The most frequently reported reasons to keep patients intubated for 5 days or longer (*n* = 48) were severe swelling (15 of 48 patients [31%]), pneumonia (5 of 48 [10%]), and macroglossia (3 of 48 [6%]).

**Table 3.** Respiratory Support and Postoperative Complications^a^

	Timing of Extubation/Tracheostomy			
	IEX	DEX	STT	LTT
No. of patients	62	162	9	42
Days intubated^b^				
0	62 (100)	—	0 (0)	13 (31)
1	—	32 (20)	3 (33)	10 (24)
2	—	42 (26)	1 (11)	7 (17)
3	—	23 (14)	1 (11)	2 (5)
>3	—	65 (40)	3 (33)	10 (23)
Respiratory support before extubation^c^				
Open tube	—	9 (6)	—	—
Oxygen	—	12 (7)	—	—
Mechanical ventilation	—	140 (86)	—	—
Respiratory support after extubation^d^				
None	38 (61)	73 (45)	0 (0)	16 (38)
Oxygen	21 (34)	80 (50)	9 (100)	26 (62)
Oropharyngeal airway	3 (5)	4 (2)	0 (0)	0 (0)
CPAP	0 (0)	5 (3)	1 (11)	1 (2)
Endotracheal reintubation	1 (2)	12 (7)	—	—
New tracheostomy	0 (0)	6 (4)	—	—
Restarting mechanical ventilation	—	—	2 (22)	3 (7)
Complications^e^				
Pneumonia	0 (0)	24 (15)	2 (22)	0 (0)
Pressure ulcer	1 (2)	9 (6)	1 (11)	2 (5)

IEX, immediate extubation; DEX, delayed extubation; STT, short-term tracheostomy; LTT, long-term tracheostomy; CPAP, continuous postoperative airway pressure.

a Values represent number of patients (percentage). Patients could have multiple types of respiratory support or complications.

b The STT and LTT columns show days of mechanical ventilation. In 1 patient in the STT group, the number of days of mechanical ventilation was unknown.

c In 1 patient, the type of respiratory support before extubation was unknown.

d The STT and LTT columns show respiratory support after stopping mechanical ventilation.

e In 1 patient in the DEX group, complications were unknown.

**Fig. 3.**
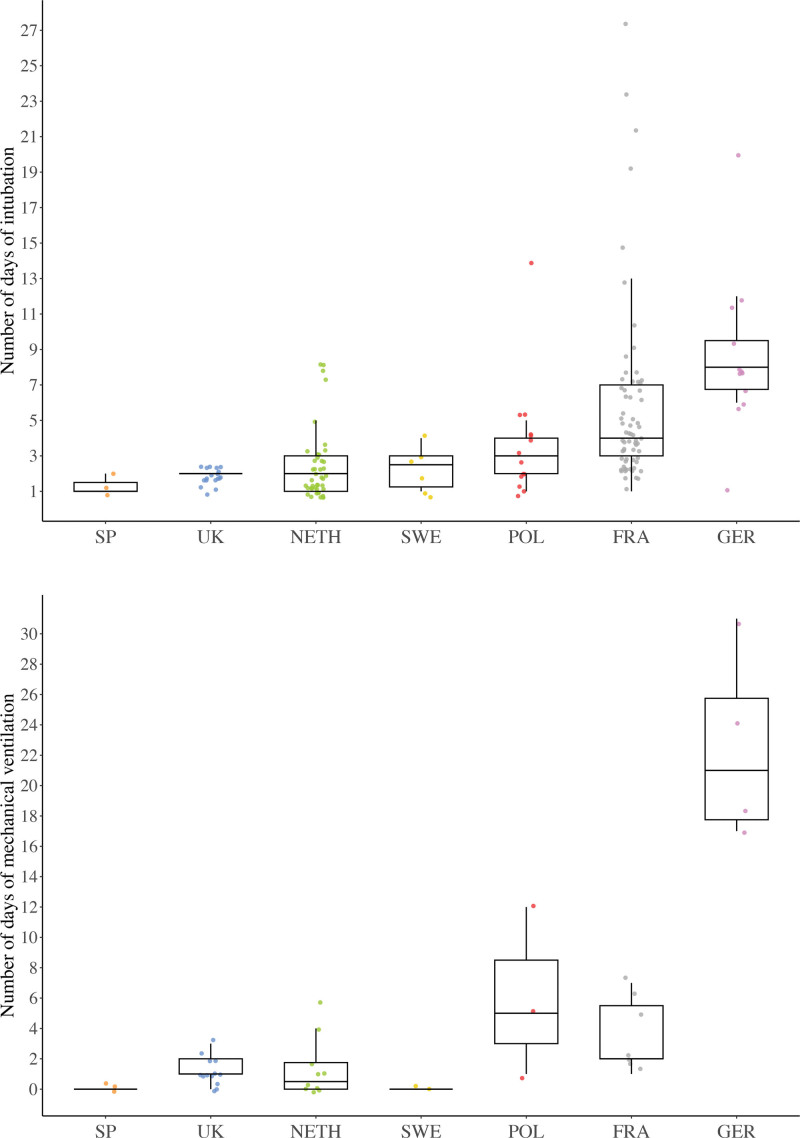
Boxplots show the number of days (median and IQR) of intubation in patients with delayed extubation (*above*) and mechanical ventilation in patients with a long-term tracheostomy (*below*) by medical center location. The *colored dots* show the distribution of patients and outliers per medical center. *SP*, Spain; *UK*, United Kingdom; *NETH*, the Netherlands; *SWE*, Sweden; *POL*, Poland; *FRA*, France; *GER*, Germany.

Although the numbers were too small to make any definitive statements, total intubation time appeared to be similar among most centers; only in the centers in France and Germany were patients possibly intubated longer (Fig. 3, *above*). Not all differences were due to patient characteristics (Table 4).

**Table 4.** Characteristics of Patients with Delayed Extubation by Medical Center

	Craniofacial Center						
	Spain	UK	Netherlands	Sweden	Poland	France	Germany
No. of patients	3	18	43	6	14	66	12
Median age (IQR), yrs	10 (8–14)	11 (7–15)	13 (7–18)	7 (7–10)	11 (8–18)	6 (3–12)	5 (4–7)
OSA classification, no. (%)							
No/mild	0 (0)	0 (0)	34 (80)	2 (33)	0 (0)	47 (71)	2 (17)
Moderate	1 (33)	3 (17)	3 (6)	0 (0)	6 (43)	12 (18)	0 (0)
Severe	2 (67)	15 (83)	6 (14)	4 (67)	8 (57)	2 (3)	0 (0)
Unknown	0 (0)	0 (0)	0 (0)	0 (0)	0 (0)	5 (8)	10 (83)
Median EBL (IQR), mL/kg	47 (27–54)	-	43 (28–72)	43 (39–43)	-	-	-
Median TBV (IQR), mL/kg	33 (20–36)	22 (11-40)	20 (6–43)	-	-	25 (16–43)	22 (19–84)

EBL, estimated blood loss; TBV, transfused blood volume.

After a monobloc procedure, patients remained intubated for a median period of 5 days (IQR, 3 to 8 days), compared with 3 days (IQR, 3 to 5 days) after a Le Fort III (Table 5).

**Table 5.** Patient Characteristics, Respiratory Support, and Postoperative Complications by Type of Surgery^a^

	Monobloc		Le Fort III	
	IEX	DEX	IEX	DEX
No. of patients (%)	38 (38)	61 (62)	24 (19)	101 (81)
Median age (IQR), yrs	6.3 (4.1–9.4)	3.0 (2.0–5.5)	15.3 (8.6–18.4)	12.1 (8.0–16.3)
OSA classification, no. (%)				
No/mild	25 (66)	33 (54)	16 (67)	52 (51)
Moderate	2 (5)	9 (15)	2 (8)	16 (16)
Severe	11 (29)	10 (16)	5 (21)	27 (27)
Unknown	0 (0)	9 (15)	1 (4)	6 (6)
Median EBL (IQR), mL/kg	53 (29–70)	84 (61–124)	27 (21–39)	36 (26–46)
Median TBV (IQR), mL/kg	22 (17–36)	38 (24–66)	7 (0–15)	16 (6–25)
Median days intubated (IQR)	—	5 (3–8)	—	3 (3–5)
Respiratory support before extubation, no. (%)				
Open tube	—	3 (5)	—	6 (5)
Oxygen	—	5 (8)	—	8 (8)
Mechanical ventilation	—	53 (87)	—	87 (86)
Respiratory support after extubation, no. (%)				
None	24 (63)	28 (46)	14 (58)	45 (45)
Oxygen	12 (32)	25 (41)	9 (38)	55 (54)
Oropharyngeal airway	2 (5)	3 (5)	1 (4)	1 (1)
CPAP	0 (0)	2 (3)	0 (0)	3 (3)
Endotracheal reintubation	1 (3)	5 (8)	0 (0)	7 (7)
New tracheostomy	0 (0)	2 (3)	0 (0)	4 (4)
Complications, no. (%)				
Pneumonia	0 (0)	10 (16)	0 (0)	14 (14)
Pressure ulcer	1 (3)	8 (13)	0 (0)	1 (1)

IEX, immediate extubation; DEX, delayed extubation; EBL, estimated blood loss; TBV, transfused blood volume; CPAP, continuous postoperative airway pressure.

a One patient could have multiple types of respiratory support or complications.

### Respiratory Support after Extubation

Fifty-eight out of 62 patients (94%) with immediate extubation required either no or only oxygen support after extubation, compared with 137 of 162 patients (85%) with delayed extubation (Table 3). In both groups, most patients only needed respiratory support for a few hours; the median number of days of respiratory support after extubation was 0 (IQR, 0 to 1 day) in both groups. A chi-square analysis of independence did not find a significant association between respiratory support after extubation and perioperative airway management (χ^2^ = 4.72, *df* = 1, *P* = 0.030). Immediate extubation was effective in patients with moderate to severe OSA, with 19 of 20 patients (95%) with immediate extubation requiring either no or only oxygen support (3 of 4 with moderate OSA and 16 of 16 [100%] with severe OSA), compared with 49 of 62 patients (79%) with delayed extubation (21of 25 [84%] with moderate OSA and 28 of 37 [76%] with severe OSA). The same appeared to be true for younger children (<5 years old): 12 of 13 patients (92%) with immediate extubation and 38 of 48 (79%) with delayed extubation required either no or only oxygen support. Likewise, immediate extubation after a monobloc procedure could be contemplated, with 35 of 38 patients (92%) with immediate extubation versus 50 of 61 (82%) with delayed extubation receiving no or only oxygen support (Table 5). The rates were similar for patients treated with a Le Fort III procedure (23 of 24 patients [96%] and 87 of 101 patients [86%], respectively).

Ultimately, 1 of 62 patients (2%) with immediate extubation and 16 of 161 (10%) with delayed extubation needed to be reintubated or receive a (new) tracheostomy postoperatively. The patient with immediate extubation needed reintubation because of obstructive breathing caused by severe postoperative swelling of the upper airway. Indications for reintubation in patients with delayed extubation were obstructive breathing caused by severe postoperative swelling in 5 of 10 patients, respiratory distress due to pneumonia in 2 of 10, accidental self-extubation in 2 of 10, and unknown cause in 1 of 10. Four patients with delayed extubation received a (new) tracheostomy postoperatively, 3 of 4 because of severe swelling and 1 of 4 due to respiratory deterioration. The tracheostomies were mostly inserted short term, but 1 patient required a long-term tracheostomy. Two patients with delayed extubation had received both endotracheal reintubation and (new) tracheostomies; 1 had an accidental self-extubation with reintubation and ultimately received a tracheostomy because of macroglossia, and the other received a tracheostomy after multiple extubation failures because of severe swelling. Their tracheostomies were removed after 2 and 8 months, respectively. The patients who needed reintubation were a little younger (median age, 6.3 years; IQR, 3 to 9 years), more often had moderate to severe OSA (64%), and were intubated longer (median, 6 days; IQR, 3 to 8 days) than the overall group.

### Respiratory Support in Patients with a Tracheostomy

Patients with short-term tracheostomies remained ventilated for 3 days (IQR, 1 to 6 days) and those with long-term tracheostomies for 1 day (IQR, 0 to 4 days). The most frequently reported indications to keep patients ventilated directly after surgery in the short- and long-term tracheostomized groups were routine management in all midface surgery (3 of 9 and 13 of 29 patients [45%], respectively) and severe OSA (2 of 9 and 8 of 29 patients [28%], respectively). Similar to intubation time in patients with delayed extubation, ventilation time in patients with long-term tracheostomies appeared to be comparable among most centers; only in the centers in Poland, France, and Germany was ventilation possibly longer (Fig. 3, *below*).

All patients with short-term tracheostomies received respiratory support after ceasing mechanical ventilation (Table 3). Twenty-six patients (62%) with long-term tracheostomies received respiratory support after ceasing ventilation. Respiratory support was only needed for a short period in both groups (1 day [IQR, 0 to 4 days] in the short-term tracheostomy group and 0 days [IQR, 0 to 1 day] in the long-term group). Two patients with short-term tracheostomies had to be recannulated or had ventilation restarted, in 1 patient because of accidental decannulation and in the other for reasons unrelated to respiratory status. Four patients with short-term tracheostomies were decannulated within 2 months after surgery, 4 were decannulated at the end of the distraction period, and 1 patient needed a long-term tracheostomy. Ventilation had to be restarted in 3 of 42 patients (7%) with long-term tracheostomies because of respiratory failure after ceasing mechanical ventilation.

### Postoperative Complications

There were no deaths related to perioperative airway management in this cohort. A significant association between timing of extubation and postoperative pneumonia was found; patients with immediate extubation were less likely to develop postoperative pneumonia compared with patients with delayed extubation (0 of 62 [0%] versus 24 of 161 [15%], respectively; χ^2^ = 8.86, *df* = 1, *P* = 0.001). Pressure ulcers developed in 1 of 62 patients (2%) with immediate extubation and 9 of 161 (6%) with delayed extubation. Two out of 9 patients with short-term tracheostomies and none with long-term tracheostomies developed postoperative pneumonia. Pressure ulcers developed in 1 of 9 patients with short-term tracheostomies and 2 of 42 patients (5%) with long-term tracheostomies. The number of patients who developed postoperative pneumonia was comparable between both procedures (Table 5) (10 of 61 patients [16%] after a monobloc procedure and with delayed extubation compared with 14 of 101 [14%] after a Le Fort III). More patients who had a monobloc procedure with delayed extubation developed pressure ulcers compared with patients who had a Le Fort III procedure (8 of 61 patients [13%] and 1 of 101 [1%], respectively).

Ultimately 36 of 275 patients (13%) developed postoperative pneumonia, pressure ulcers, or both. The analysis found an odds ratio of 1.21 (95% CI, 1.12 to 1.31) of intubation/mechanical ventilation in intubation-related complications. This means the odds of developing intubation-related complications increased by 21% with every extra day of intubation/mechanical ventilation. The Hosmer–Lemeshow test showed a good model fit (χ^2^ = 8.35, *df* = 8, *P* = 0.4). Overall postoperative complications are reported in Table 6.

**Table 6.** Postoperative Complications Overall^a^

	Total (*n* = 275)	Monobloc (*n* = 129)	Le Fort III (*n* = 146)
Pneumonia	26 (9)	11 (9)	15 (10)
Pressure ulcer	13 (5)	11 (9)	2 (1)
Wound infection	26 (9)	18 (14)	8 (5)
Meningitis	3 (1)	1 (1)	2 (1)
Eye cellulitis/keratitis	12 (4)	6 (5)	6 (4)
Corneal ulcer	7 (3)	3 (2)	4 (3)
Cerebrospinal fluid leak	61 (22)	46 (36)	15 (10)
Distraction material migration	14 (5)	6 (5)	8 (5)

a Values represent number of patients (percentage). Postoperative complications were unknown in 1 patient.

## DISCUSSION

This study aimed to lay the scientific foundation for perioperative airway management following midface advancements in syndromic craniosynostosis and to investigate the possibility of immediate extubation. Perioperative airway management differed among the craniofacial centers of expertise. Delayed extubation was the most frequently used method in most centers, and only in 2 centers was immediate extubation the current standard of care. The use of short-term tracheostomies was limited and was reserved for selected cases in most centers, given their invasive nature.

In most centers, the current choice for the initial type of perioperative airway management, apart from long-term tracheostomies, was mostly independent of clinical factors, such as OSA, and based primarily on tradition. Virtually all children remained intubated after surgery, except for in the centers in Spain and the Netherlands. Whether the routine use of short-term tracheostomies will be of value remains to be seen with the change in protocol within the United Kingdom.

Our primary analysis showed no association between the number of patients requiring respiratory support after extubation and the timing of extubation, indicating that immediate extubation following midface surgery is a safe option. However, a clear benefit of immediate extubation was found regarding postoperative pneumonia; patients who had received immediate extubation were less likely to develop postoperative pneumonia than those who had received delayed extubation. Immediate extubation is likely an option in young children or patients with moderate to severe OSA, as 92% and 95% of them, respectively, needed no or only noninvasive respiratory support after extubation. Prolonged endotracheal intubation, with or without mechanical ventilation, has distinct disadvantages. It can lead to immobility-associated prolonged periods of swelling and pressure ulcers^15,17^; it is a risk factor for postoperative stridor and pneumonia^14,16^; and in some cases, the tube can impede the normal blood circulation of the tongue, which can initiate or maintain an airway obstruction.^18^ Our secondary analysis is in line with the literature and showed that every extra day of intubation/mechanical ventilation increased the odds of developing pneumonia or pressure ulcers by 21%.

Tracheostomies, as with prolonged intubation, also have disadvantages, including requiring extra surgery, with accompanying postoperative complications, to place or remove the tracheostomy^19,20^; misplacement of the tracheostomy tube, secretions, or blood clots, which can potentially block the tracheostomy^19^; the complexity of care for patients with tracheostomies and the need for trained nurses and parents^21^; the psychological effect of a noticeable scar on patients; and the rare but severe complication of long-term tracheal stenosis. An additional potential advantage of immediate over delayed extubation and short-term tracheostomies is the possible reduction of the length of stay in the pediatric ICU. The number of pediatric ICU beds is usually restricted due to high costs and the need for experienced staff. Length of stay in the pediatric ICU is the most significant contributor to total pediatric ICU cost.^22,23^ The indication for ICU-level care is, among other factors (eg, hospital nursing protocols), influenced by the type of airway management. Changing the type of airway could, therefore, potentially reduce the length of stay.

For many years, extubation has been delayed for several days on the assumption that the anatomically abnormal upper airway in combination with midface surgery would lead to functional obstruction immediately postoperatively. The clinical results of this study show that this belief is unwarranted and that delayed extubation should not be indicated as standard practice. Taking these findings together, we recommend the following guidelines for making an SOP in airway management after midface advancements:

1. Immediate extubation should be considered routine management in patients with no or mild OSA.
2. Immediate extubation should also be the aim in patients with moderate to severe OSA. An attempt at extubation should be contemplated at the end of the procedure, given the odds ratio finding of 1.21 for 1 extra day of intubation on intubation-related complications and because extubation in the operating room by the treating anesthesiologist creates the safest conditions for such a procedure. The following additional factors should be considered: always perform upper airway endoscopy before the midface surgery, and perform additional adenoid, tonsil, or adenotonsillectomy procedures when feasible. In carefully selected cases, this could be considered during the midface advancement procedure but preferably not, as this could add to airway challenges. In case of concomitant airway obstruction at the tongue-base level, some degree of OSA will persist postoperatively and could influence the timing of extubation. Finally, consider decreasing the degree of OSA postoperatively by directly starting distraction on the operating table to increase the advancement during surgery. In line with this previous concern is the need for postoperative continuance of continuous postoperative airway pressure (CPAP) therapy and any difficulties fitting the CPAP mask in combination with an external frame. The earlier-mentioned methods to reduce the persistence of postoperative OSA are thus also essential to limit the need to continue postoperative CPAP. In other words, anticipate the degree of OSA that might persist and the need to continue preceding respiratory support postoperatively.
3. Immediate extubation can also be contemplated in young children after considering the above-mentioned factors and after the craniofacial team’s comprehensive assessment of the individual patient.
4. Case-specific factors, including prematurity, patient weight, congenital heart disease, intraoperative blood loss, severity of OSA, and presence of multilevel airway obstructions (particularly of the tongue base), can be indications for delayed extubation and should thus be taken into account when deciding on the timing of extubation. Administration of corticosteroids can be contemplated in the perioperative setting to reduce (laryngeal) edema.^24–26^
5. If the decision is made for delayed extubation, consider extubation within the first 24 hours. This decision should be made following a multidisciplinary consultation.

### Limitations

Our study has several limitations. First, information on operating times was not collected as a covariate. Surgical injury and stress bring on a sequence of systemic responses, ultimately affecting the redistribution of fluid between the intravascular and interstitial spaces in the inflammatory phase of wound healing, which is clinically noticed as swelling or edema. Therefore, prolonged operative duration can be associated with more postoperative swelling and a higher risk of respiratory complications. Furthermore, only information on intraoperative transfused red blood cells was collected; information on postoperative transfusions or other perioperative fluids (eg, crystalloid or colloid) was not collected. The total amount of fluid administered and the ratio between transfused blood volume and other fluids are highly dependent on the anesthesiologist’s preference. Therefore, it would likely be different among hospitals and patients. Excessive fluid administration can lead to fluid accumulation in the lungs and may predispose patients to respiratory failure or pneumonia. In future prospective studies, particular emphasis should be placed on establishing standardized definitions for all pertinent perioperative parameters, including fluid resuscitation, airway grade, time in the operating room, and corticosteroid use. These studies should aim to investigate how these variables affect airway management in midface surgical procedures.

## CONCLUSIONS

This study lays the foundation for SOPs for perioperative airway management following midface surgery in syndromic craniosynostosis. Immediate extubation following midface surgery is demonstrated to be a safe option that is associated with a minimal need for additional airway support and few complications. Further investigation of its implementation should examine individual patient selection, especially with regard to levels of airway obstruction in moderate to severe OSA.

## DISCLOSURE

The authors have no conflicts of interest to report.

## ACKNOWLEDGMENTS

This work is generated within the European Reference Network (ERN) for rare and/or complex craniofacial anomalies and ear, nose, and throat disorders (ERN CRANIO). ERN CRANIO is co-funded by the European Union.

## Supplementary Material


