# Supplementary material for: A European Multicenter Outcome Study of Perioperative Airway Management Policies following Midface Surgery in Syndromic Craniosynostosis
Source: Plast Reconstr Surg. 2024 Jan 30;154(6):1281–92. doi: 10.1097/PRS.0000000000011317 (PMC11584185; doi:10.1097/PRS.0000000000011317)
Supplement: Supplementary file 1 [file prs-154-01281-s001.pdf]

Appendix, Supplemental Digital Content 1

Multiple imputation for missing data

Multiple-imputation-by-chained equation (MICE package) in R was used to handle missing values and was based on the method described by van Buuren et al.. The accompanying table gives a summary of the proportion of missing cases and number of missing values per variable. The percentage of missing values ranged between 0% and 56% across the variables. One hundred five out of the 275 (38%) records were incomplete. Missing data pattern was assumed to be missing at random (MAR). Most missing data were in the predictor variables, only in one patient the outcome variable was missing. Most missing values were in estimated blood loss (EBL) and obstructive sleep apnea (OSA) classification. EBL was missing in the centers in the United Kingdom, Germany, and France because only blood transfusion is documented after surgery. In the centre in Poland neither EBL nor blood transfusion could be recovered from the patient files. In Sweden EBL was documented. Most missing values for OSA classification were in Germany since polysomnography was routinely performed at an external facility, limiting the availability of exact OSA classification.

Table. Missing values per variable

Variable	# of patients	% of patients
Study center	0	0
Age	0	0
Gender	0	0
Diagnosis	0	0
Indication surgery	0	0
Type of surgery	0	0
Type of perioperative airway management	0	0

Type of respiratory support	0	0
Days of intubation	1	0
Intubation related complications	1	0
Days of respiratory support	2	1
OSA present	13	5
Weight	17	6
Classification OSA	19	7
Transfusion	43	16
Transfusion per kg	43	16
Blood loss	154	56
Blood loss per kg	154	56

For the numeric variables the 'predictive mean matching method' was used, for factor columns with two categories the 'logistic regression method', and for columns with more than two categories the 'polytomous logistic regression method'. Passive imputation was used to impute Blood loss per kg by the function 'Blood loss/Weight', and Transfusion per kg by 'Transfusion/Weight'. Care was taken to avoid multicollinearity; Blood loss, Transfusion, and Weight were only used as predictor variables for Blood loss, Transfusion, and Weight. For the other variables, Blood loss per kg and Transfusion per kg were used as predictor variables. Age was not used to impute Weight due to collinearity. Ultimately, 50 imputed datasets were created and analyzed. Convergence was checked and reached. Imputed values were checked to be realistic. Density plots were plotted to check the distribution of the imputed values against the observed values. After multiple imputation, the logistic regression model used for the secondary analysis was applied to each imputed dataset with the '*with*' function. The pooled odds ratio and corresponding 95% confidence interval from the secondary analysis were then gathered by the '*pool*' function.
