# Supplementary material for: A European Multicenter Outcome Study of Perioperative Airway Management Policies following Midface Surgery in Syndromic Craniosynostosis
Source: Plast Reconstr Surg. 2024 Jan 30;154(6):1281–92. doi: 10.1097/PRS.0000000000011317 (PMC11584185; doi:10.1097/PRS.0000000000011317)
Supplement: Supplementary file 2 [file prs-154-01281-s002.pdf]

Supplemental Digital Content 2

Table 1. Summary of center specific considerations/protocols in airway management following midface advancement

**Craniofacial center**	**IEX - DEX**	**STT - LTT**	**Start distraction during surgery**
**SP**	- Since 10 years has IEX been protocol<br>- Indications DEX: severe swelling, hemodynamic instability, or another medical problem<br>- In case of DEX: extubation always within 6 hours	- STTs are not used<br>- LTTs are used in case of severe OSA	+
**NETH**	- Before 2010, all patients received DEX for 1–3 days<br>- Between 2010-2015 indications for DEX were mainly severe airway swelling or excessive EBL<br>- Since 2015, have all patients received IEX	- STTs are not used<br>- LTTs are used in case of severe respiratory problems in young children (< 2 years old)	+
**SWE**	- Decisions about airway management are always made after multidisciplinary consultation	- STTs are also used after taking into consideration aforesaid factors	+

	- Assumed postoperative swelling, EBL, the patients history and the fact that the postoperative care takes place on an adult ICU are taken into consideration		
**UK**	- Before 2005, most children received DEX for 1-3 days	- Currently, the unit has moved to STTs regularly and STTs are inserted 2 weeks before midface surgery<br>- Patients are ventilated overnight if prolonged surgery or massive EBL	+
**GER**	- Nearly all children receive DEX<br>- Extubation depends on postoperative swelling and is usually attempted on days 2-5 postoperatively	- STTs are not routinely used but would be considered for very young children with pre-existing severe airway obstruction	-
**POL**	- Started performing LF3 and MB procedures in 2012<br>- All children remain intubated for a minimum of 2-3 days	- Midface advancements are not performed before age 7<br>- LTTs are placed in case of severe OSA in children < 7 years old	-
**FRA**	- Discussion on extubation from day 2 postoperatively and always after a postoperative CT	- The use of STTs is limited<br>- LTTs are used in young children with severe OSA or children with tracheal anomalies	+

	- Extubation is based on local edema, fever, CSF leaks, intracranial complications, and response to a decrease in sedation

IEX: immediate extubation, DEX: delayed extubation, STT: short term tracheostomy, LTT: long term tracheostomy. SP: Spain, NETH: Netherlands, SWE: Sweden, UK: United Kingdom, GER: Germany, POL: Poland, FRA: France
